# Comparative Metabolome and Transcriptome Analysis Reveals the Defense Mechanism of Chinese Cabbage (*Brassica rapa* L. ssp. *pekinensis*) against *Plasmodiophora brassicae* Infection

**DOI:** 10.3390/ijms251910440

**Published:** 2024-09-27

**Authors:** Xiaochun Wei, Yingyi Du, Wenjing Zhang, Yanyan Zhao, Shuangjuan Yang, Henan Su, Zhiyong Wang, Fang Wei, Baoming Tian, Haohui Yang, Xiaowei Zhang, Yuxiang Yuan

**Affiliations:** 1 Institute of Vegetables, Henan Academy of Agricultural Sciences, Graduate T&R Base of Zhengzhou University, Zhengzhou 450002, China; jweixiaochun@126.com (X.W.); 18860369504@163.com (Y.D.); zhangwj050@163.com (W.Z.); zhaoyanyan9621@163.com (Y.Z.); sjyang_0614@163.com (S.Y.); 18810835083@163.com (H.S.); nkywzy@163.com (Z.W.); fangwei@zzu.edu.cn (F.W.); tianbm@zzu.edu.cn (B.T.); yanghaohui127@163.com (H.Y.); 2 School of Agricultural Sciences, Zhengzhou University, Zhengzhou 450001, China

**Keywords:** Chinese cabbage, metabolome, transcriptome, *Plasmodiophora brassicae*, phenylpropanoid, arachidonic acid

## Abstract

Chinese cabbage (*Brassica rapa* L. ssp. *pekinensis*) ranks among the most cultivated and consumed vegetables in China. A major threat to its production is *Plasmodiophora brassicae,* which causes large root tumors, obstructing nutrient and water absorption and resulting in plant withering. This study used a widely targeted metabolome technique to identify resistance-related metabolites in resistant (DH40R) and susceptible (DH199S) Chinese cabbage varieties after inoculation with *P. brassicae*. This study analyzed disease-related metabolites during different periods, identifying 257 metabolites linked to resistance, enriched in the phenylpropanoid biosynthesis pathway, and 248 metabolites linked to susceptibility, enriched in the arachidonic acid metabolism pathway. Key metabolites and genes in the phenylpropanoid pathway were upregulated at 5 days post-inoculation (DPI), suggesting their role in disease resistance. In the arachidonic acid pathway, linoleic acid and gamma-linolenic acid were upregulated at 5 and 22 DPI in resistant plants, while arachidonic acid was upregulated at 22 DPI in susceptible plants, leading to the conclusion that arachidonic acid may be a response substance in susceptible plants after inoculation. Many genes enriched in these pathways were differentially expressed in DH40R and DH199S. The research provided insights into the defense mechanisms of Chinese cabbage against *P. brassicae* through combined metabolome and transcriptome analysis.

## 1. Introduction

Chinese cabbage (*Brassica rapa* L. ssp. *pekinensis*), which originated in China, is a biennial herbaceous plant of the *B. rapa* subspecies. It is an important cruciferous vegetable widely cultivated globally, especially in East Asia, and serves as a major food and economic crop. Chinese cabbage is highly valued for its high yield, rich nutritional value, and adaptability to various climatic conditions. However, Chinese cabbage production still faces numerous biotic and abiotic stressors, among which clubroot disease is a serious soilborne disease that significantly affects Chinese cabbage growth and yield. Clubroot disease is caused by *Plasmodiophora brassicae*, a pathogenic fungus that infects the roots of cruciferous plants. After infection, the roots develop tumor-like swellings, severely affecting water and nutrient absorption and ultimately leading to stunted growth, leaf yellowing, wilting, and even death. Clubroot disease is widespread globally, especially in environments with cool and moist soil conditions. It has complex transmission routes and is highly difficult to control, posing significant challenges to agricultural production. Currently, there were two internationally recognized *P. brassicae* classification systems: the Williams system [1] and the European clubroot differential (ECD) system [2]. The Williams system classified *P. brassicae* into 16 pathotypes based on the resistance performance of four hosts, while the ECD system used five hosts each from Chinese cabbage, turnip, and cabbage to classify the pathogen [2]. The main *P. brassicae* isolate in China is pathogen isolate 4, as identified by the Williams system. Yuan, et al. [3] achieved a more precise differentiation of a more pathogenic variant of *P. brassicae* pathogen isolate 4 using the ECD system.

Clubroot disease resistance primarily originates from the A genome of European turnip, which is mainly controlled by single dominant genes. To date, 36 loci conferring clubroot resistance have been identified in the A genome, distributed across chromosomes A01, A02, A03, A05, A06, A07, and A08: the loci *CR6a*, *Crr2*, and *PbBa1.1* on chromosome A01 [4]; *CRc* and *Rcr8* on chromosome A02 [5]; *CRa*, *CRb*, *CRd*, *CRk*, *CRq*, Crr3, *Rcr1*, *Rcr2*, *Rcr4*, *Rcr5*, *BraA.CRa*, *BraA.CRc*, *PbBa3.1*, *PbBa3.2*, *PbBa3.3*, *CRb^kato^*, *CR6b*, *BraA3PSX.CRa/b^kato^1.1*, and *BraA3PSX.CRa/b^kato^1.2* on chromosome A03 [6]; *CrrA5* on chromosome A05 [7]; *Crr4* on chromosome A06 [8]; *qBrCR38-1* on chromosome A07 [9]; and *CRs*, *Crr1a*, *Rcr3*, *Rcr9*, *PbBa8.1*, *BraA.CRb*, *qBrCR38-2*, *PbBrA08^Banglim^*, *Rcr9wa*, and *PbCRA8.1.6* on chromosome A08 [10].

Metabolomics is a method that reveals the physiological state and stress responses of an organism by analyzing changes in all small-molecule metabolites. These metabolic changes not only reflect the response mechanism of plants to pathogen infection but also provide potential biomarkers for early diagnosis and disease monitoring. Lan, et al. [11] found that resistant and susceptible Chinese cabbage genotypes showed distinct phytohormone levels and metabolome changes with increased salicylic acid levels and specific metabolite accumulations linked to clubroot resistance. Wei, et al. [12] investigated transcriptomic and metabolomic changes in *Brassica* roots during *P. brassicae* infection and identified key genes and pathways in resistant and susceptible plants, highlighting hormone-mediated defense mechanisms for improved clubroot resistance in *B. rapa*.

Transcriptomics research has also provided important gene expression information for understanding clubroot disease. Transcriptomics has revealed the gene regulatory mechanisms of plants during *P. brassicae* infection by analyzing changes in gene expression levels. Additionally, transcriptomics analysis has identified some key disease resistance genes and regulatory factors, providing new targets and strategies for breeding. Siemens, et al. [13] highlighted the roles of auxin and cytokinin in disease progression. Transcriptome and proteome data have linked glucosinolate and auxin pathways to gall development, with variations between *Brassica* and *Arabidopsis* [14]. Using laser microdissection and pressure catapulting (LMPC), Schuller, et al. [15] isolated infected cells to study pathogen stages and host transcriptional changes, revealing the upregulation of auxin, cytokinin, and brassinosteroid genes. The *Rcr1* gene and markers were crucial for breeding resistant canola, and identifying differentially expressed genes (DEGs) helped to explain clubroot resistance pathways, aiding durable resistance strategies [16]. Investigating glucosinolates (GSLs) in clubroot disease revealed that resistant *Matthiola incana* limits cortical infection, while susceptible *Brassica napus* accumulates aliphatic GSLs. Jasmonic acid (JA) enhanced GSLs, with aromatic GSLs defending *M. incana,* and aliphatic GSLs, regulated by *BnMYB28.1*, playing a role in the secondary infection stage in *B. napus* [17]. Wei, et al. [12] studied *P. brassicae* infection in *Brassica* crops, particularly Chinese cabbage, and revealed distinct phytohormone levels and transcriptomic changes in resistant and susceptible genotypes, highlighting key defense mechanisms and pathways involved in clubroot resistance. Wei, et al. [18] comprehensively analyzed the transcriptome, small RNAs, degradome, and phytohormones in resistant and susceptible *B. rapa* lines to explore the infection mechanism of *P. brassicae*; identified key genes involved in the auxin, cytokinin, jasmonic acid, and ethylene cycles; and proposed a regulatory model of plant hormones during critical infection periods. Liao, et al. [19] demonstrated that silencing *bra-miR167a* in *Arabidopsis* enhanced clubroot resistance by promoting lateral root development, increasing auxin levels, and upregulating disease resistance-related genes, providing new insights into the molecular mechanisms resulting in clubroot resistance. Transcriptomic analysis in Chinese cabbage identified transcription factors such as *bHLH*, *WRKY*, and *NAC* that were pivotal in primary and secondary infection, highlighting genes involved in plant defense, biosynthesis, and carbohydrate transport that were crucial for managing clubroot disease in *Brassica* crops [20]. Liu, et al. [21] used metagenomic sequencing to analyze rhizosphere microbiota after clubroot infection and identified microbial community changes and functional impacts, revealing that oxidative phosphorylation and glycerol-1-phosphatase are crucial for enhancing resistance. Oh, et al. [22] investigated Akimeki’s early molecular responses to two *P. brassicae* strains using RNA sequencing, highlighting genes involved in defense and immunity.

In addition to metabolomics and transcriptomics, proteomics plays a crucial role in clubroot disease research. Proteomics have identified and quantified intracellular proteins and explored their roles in disease processes. Functional analysis of these proteins not only helps to uncover the pathological mechanisms of root diseases but also provides a theoretical basis for developing new prevention and control measures. Clubroot, which is caused by *P. brassicae*, affects canola in Alberta, Canada. Protein analysis after inoculation revealed significant changes, including cytokinin and lignin-related proteins, impacting early infection stages and host susceptibility to the pathogen [23]. Su, et al. [24] identified 295 differentially expressed proteins in Chinese cabbage infected with *P. brassicae*, highlighting the role of brassinosteroid metabolism and the cycloartenol synthase enzyme in disease development using high-throughput functional proteomics analysis. Ji, et al. [25] evaluated *B. rapa* seedlings infected with *P. brassicae* and identified numerous differentially expressed proteins, highlighting significant roles in salicylic acid and jasmonic acid/ethylene-mediated resistance; Gene Ontology (GO) and Kyoto Encyclopedia of Genes and Genomes (KEGG) analysis showed the involvement of plant defense and signaling pathways.

With the rapid advancement of bioinformatics, integrative multi-omics analysis has provided an opportunity and a clear technical roadmap to comprehensively explain the interactions between plants and pathogens. Although there have been some studies on the omics analysis of clubroot disease in Chinese cabbage, there has been a lack of in-depth research on the joint analysis of transcriptomics and metabolomics of clubroot disease. This study aimed to explore the defense mechanisms utilized by Chinese cabbage against clubroot disease using integrated metabolomics and transcriptomics analysis.

## 2. Results and Analysis

### 2.1. Identification of Resistance to Clubroot Disease in Chinese Cabbage

**Figure 1:**
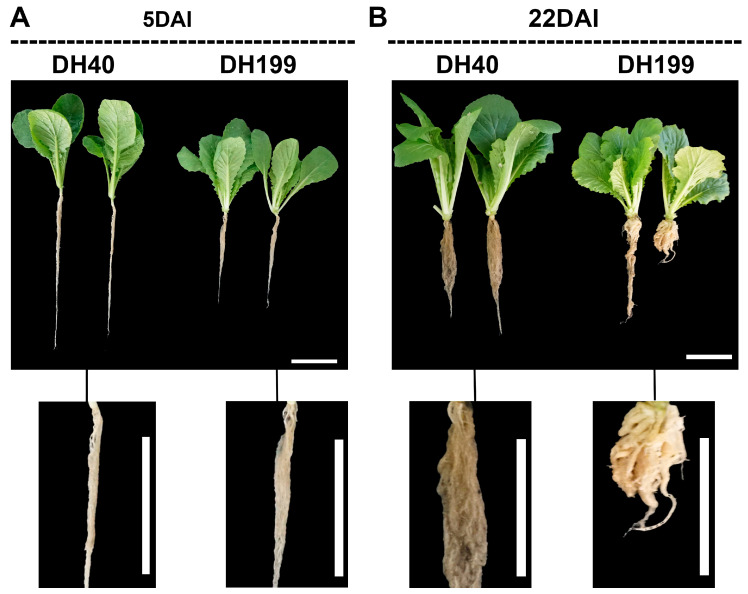
Representative images of DH40 and DH199 materials at 5 and 22 DAI (days after inoculation) of *P. brassicae*. Bar = 5 cm. (**A**) 5 DAI. (**B**) 22 DAI.

Morphological observations of resistant Chinese cabbage doubled haploid (DH) lines DH40R and susceptible Chinese cabbage DH199S inoculated with *P. brassicae* revealed that neither DH40R nor DH199S showed any small tumors on their main roots at 0 and 5 days post-inoculation (DPI). At 22 DPI, the main and lateral roots of DH40R exhibited no abnormalities, indicating complete resistance, while the main root of DH199S showed abnormal swelling, indicating susceptibility (Figure 1A,B).

### 2.2. Metabolome Results and Analysis

#### 2.2.1. Principal Component Analysis and Differentially Accumulated Metabolites in All Samples

**Figure 2:**
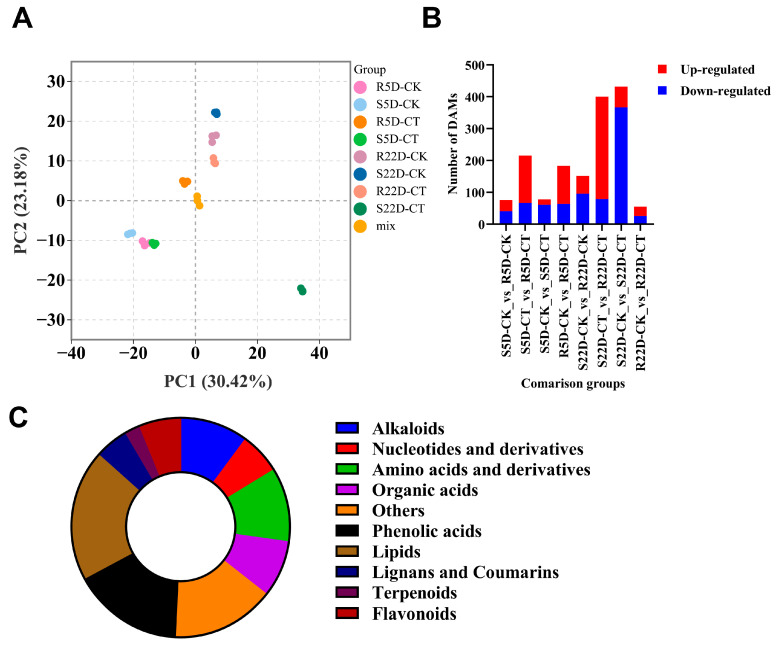
Principal component analysis (PCA), categorization, and number of metabolites in various groups. (**A**) PCA score plot. (**B**) Bar graph of the number of differentially accumulated metabolites upregulated and downregulated in different comparisons (S5D-CK_vs_R5D-CK, S5D-CT_vs_R5D-CT, S5D-CK_vs_S5D-CT, R5D-CK_vs_R5D-CT, S22D-CK_vs_R22D-CK, S22D-CT_vs_R22D-CT, S22D-CK_vs_S22D-CT, and R22D-CK_vs_R22D-CT). For each comparison group, the preceding group served as the control group. (**C**) Pie chart by classification of differentially accumulated metabolites.

DH40R and DH199S were subjected to widely targeted metabolomics sequencing at 5 and 22 DPI, with a control group for baseline comparison. Principal component analysis (PCA) revealed high intra-group cohesion and clear inter-group data segregation, indicating the reliability of the metabolomics data (Figure 2A).

There were certain differences in the metabolites accumulated in the S and R lines without clubroot inoculation. Compared to the S line with clubroot inoculation, the R line with clubroot inoculation had many relatively upregulated differentially accumulated metabolites (DAMs), indicating intrinsic metabolic differences. After stress treatment, the differences became more pronounced. These DAMs might explain the difference in clubroot resistance between the R and S lines (Figure 2B) Widely targeted metabolomics analysis identified 831 metabolites, including alkaloids (84 types, 10.11%), amino acids and derivatives (91 types, 10.95%), flavonoids (51 types, 6.14%), lignans and coumarins (41 types, 4.93%), lipids (162 types, 19.49%), nucleotides and derivatives (51 types, 6.14%), organic acids (70 types, 8.42%), others (126 types, 15.16%), phenolic acids (136 types, 16.37%), and terpenoids (19 types, 2.29%) (Figure 2C, Supplementary Table S1).

#### 2.2.2. Differential Metabolites between the Disease-Resistant Group and the Control Group

Comparative metabolomics analysis between the resistant and control groups showed that resistant Chinese cabbage significantly induced the upregulation of some alkaloids, phenolic acids, lignans and coumarins, and organic acids early after clubroot inoculation. Compared to the control group, the resistant group had 183 DAMs at 5 DPI, of which 119 were upregulated (Supplementary Figure S1A). The top 10 significantly upregulated metabolites included alkaloids (isoquinoline), lipids (LysoPC 16:2), phenolic acids (sinapaldehyde-4-O-glucoside, syringin, and 4-methoxycinnamic acid*), lignans and coumarins (7-hydroxy-6-methoxycoumarin), organic acids (citraconic acid), and others (acetosyringone, 2-(2-phenylethyl) chromone*, and 8-(benzyloxy)-1-naphthol*). Of the metabolites, 64 were downregulated (Supplementary Figure S1A), with the top 10 including amino acids and derivatives (L-glycyl-L-isoleucine*, N-glycyl-L-leucine*, and (2S,3R,4S)-4-hydroxyisoleucine*), organic acids (3-hydroxybutyric acid), alkaloids (salicylamide, N-glucosyl-p-coumaroylputrescine), others (piceatannol-3′-O-glucoside), flavonoids (5-hydroxy-6,7,8,3′,4′-pentamethoxyflavone), and phenolic acids (methyleugenol, ethyl rosmarinate) (Supplementary Table S2, R5D-CK_vs_R5D-CT). In the comparison group “R5D-CK_vs_R5D-CT”, 183 DAMs were significantly enriched in phenylpropanoid biosynthesis, indole alkaloid biosynthesis, starch and sucrose metabolism, galactose metabolism, and other pathways (Supplementary Figure S1B).

Compared to the control, the R group at 22 DPI contained 55 DAMs, of which 29 showed an increase (Supplementary Figure S1C). The notably elevated metabolites comprised alkaloids (N-hydroxypipecolic acid), phenolic acids (hydrangeifolin I, 3-O-p-coumaroylquinic acid*, and 5-O-p-coumaroylquinic acid*), flavonoids (nepetin-7-O-glucoside (nepitrin), rhamnetin-3-O-glucoside, kaempferol-3-O-galactoside-4′-O-glucoside, and luteolin-7,3′-di-O-glucoside), and others (4-hydroxyindol-3-ylmethyl glucosinolate and 4-hydroxy-3-indolylmethyl glucosinolate). Twenty-six DAMs were downregulated (Supplementary Figure S1C), among which the most significantly reduced included amino acids and derivatives (L-glycyl-L-isoleucine*), phenolic acids (3-aminosalicylic acid and 3-hydroxycinnamic acid), and terpenoids (pomolic acid*, hederagenin*, maslinic acid*, corosolic acid*, alphitolic acid*, 2-hydroxyoleanolic acid*, and euscaphic acid*) (Supplementary Table S2, R22D-CK_vs_R22D-CT). In the comparison group “R22D-CK_vs_R22D-CT”, 55 DAMs were significantly enriched in phenylpropanoid biosynthesis, indole alkaloid biosynthesis, and stilbenoid, diarylheptanoid, and gingerol biosynthesis (Supplementary Figure S1D).

#### 2.2.3. Differential Metabolites between the Susceptible and Control Groups

At 5 DPI with *P. brassicae*, there were 78 DAMs, with 17 upregulated and 61 downregulated, compared to the control group (Supplementary Figure S1E). The most significantly upregulated metabolites included flavonoids (quercetin-3-O-rutinoside (rutin), quercetin-3-O-(2″-O-rhamnosyl) galactoside, quercetin-3-O-neohesperidoside, quercetin-3-O-glucoside-7-O-rhamnoside*, quercetin-7-O-rutinoside*, quercetin-3-O-sophoroside (baimaside), kaempferol-3,7-O-diglucoside, and kaempferol-3-O-galactoside-4′-O-glucoside), and others (erythorbic acid, isoascorbic acid, and D-glucurono-6,3-lactone). These significantly upregulated flavonols, vitamins, saccharides, and alcohols were more likely associated with the susceptibility of the S line. The most significantly downregulated metabolites included lipids (LysoPC 16:2, LysoPC 18:4), amino acids and derivatives (L-cyclopentylglycine), phenolic acids (4-hydroxy-3-methoxymandelate, methyleugenol, and trans-5-O-(p-coumaroyl) shikimate), and organic acids (δ-guanidinovaleric acid, 2-hydroxybutyric acid*, 2-hydroxyisobutyric acid*, and oxalic acid) (Supplementary Table S2, S5D-CK_vs_S5D-CT). In the comparison group “S5D-CK_vs_S5D-CT”, 78 DAMs were significantly enriched in phenylpropanoid biosynthesis, flavone and flavonol biosynthesis, and the biosynthesis of unsaturated fatty acids (Supplementary Figure S1F).

Compared to the control group at 22 DPI, the S group contained 432 DAMs, of which 65 were upregulated and 367 were downregulated (Supplementary Figure S2A). The most significantly upregulated metabolites included vitamins (erythorbic acid and isoascorbic acid), phenolic acids (sinapaldehyde-4-O-glucoside, dihydrocaffeic acid, 3-nitrophenol, and hydrangeifolin I), amino acids and derivatives (L-cysteinyl-L-glycine and N-acetyl-L-methionine), nucleotides and derivatives (1-methylguanidine), and organic acids (phenylpyruvic acid and 3-guanidinopropionic acid). The most significantly downregulated metabolites included lipids (myristic acid), amino acids and derivatives (L-arginine, N-glycyl-L-leucine*, and L-cysteine), organic acids (4-acetamidobutyric acid), others (2-hydroxy-2-methylbutylglucosinolate and 2-(2-hydroxy-2-phenylethyl) chromone), alkaloids (N,N-dimethylformamide and methyl L-pyroglutamate), and phenolic acids (3,4-dihydroxybenzoic acid (protocatechuic acid)*) (Supplementary Table S2, S22D-CK_vs_S22D-CT). In comparison group “S22D-CK_vs_S22D-CT”, 432 DAMs were significantly enriched in arachidonic acid metabolism, phenylpropanoid biosynthesis, nucleotide metabolism, and thiamine metabolism (Supplementary Figure S2B).

#### 2.2.4. Differential Metabolites between Resistant and Susceptible Groups

To identify disease-resistant compounds significantly increased by clubroot infection, a comparison between the disease-resistant and -susceptible groups at 5 DPI with *P. brassicae* revealed that 3-indolylmethyl glucosinolate, isoquinoline, LysoPC 16:2, N,N-dimethylformamide, LysoPC 18:4, scopoletin (7-hydroxy-6-methoxycoumarin), 3,4-dihydroxy-L-phenylalanine (L-Dopa), N′-p-coumaroylagmatine-glucoside, 2′-deoxyinosine-5′-monophosphate, and 4-hydroxyphenylacetic acid were upregulated. In contrast, N-glycyl-L-leucine, L-glycyl-L-isoleucine, (2S,3R,4S)-4-hydroxyisoleucine, 3-hydroxybutyric acid, salicylamide, 3-hydroxycinnamic acid, 3,5,7,3′4′-pentamethoxyflavone, 3′,4′,5′,5,7-pentamethoxyflavone, ethyl rosmarinate, and quercetin-3-O-(2″-O-rhamnosyl)galactoside were significantly downregulated. Overall, in disease-resistant materials, early clubroot infection strongly induced lipids, alkaloids, lignans and coumarins, phenolic acid, amino acids and derivatives, and nucleotides and derivatives (Supplementary Figure S2C; Supplementary Table S2, S5D-CT_vs_R5D-CT). In comparison group “S5D-CT_vs_R5D-CT”, 215 DAMs were significantly enriched in indole alkaloid biosynthesis, phenylpropanoid biosynthesis, tyrosine metabolism, galactose metabolism, and other pathways (Supplementary Figure S2D).

When screening for resistance metabolites in late-stage disease, a comparison of diseased and healthy groups inoculated with *P. brassicae* for 22 DPI revealed significant upregulation of L-arginine, myristic acid, L-cysteine, putrescine, 2-hydroxy-2-methylbutylglucosinolate, N,N-dimethylformamide, 4-acetamidobutyric acid, scopoletin (7-hydroxy-6-methoxycoumarin), methyl L-pyroglutamate, and levopimaric acid in the resistant group. In contrast, cycloleucine, erythorbic acid, isoascorbic acid, dihydrocaffeic acid, L-glycyl-L-isoleucine, L-tartaric acid, 3-hydroxybutyric acid, oxalic acid, 5-hydroxy-6,7,8,3′,4′-pentamethoxyflavone, and 1-methylpiperidine-2-carboxylic acid were significantly downregulated. In summary, in the resistant materials, late-stage *P. brassicae* infection strongly induced organic acids, nucleotides and derivatives, phenolic acids, and amino acids and derivatives (Supplementary Figure S2E; Supplementary Table S2, S22D-CT_vs_R22D-CT). In comparison group S22D-CT_vs_R22D-CT, 400 DAMs were significantly enriched in Indole alkaloid biosynthesis, Arachidonic acid metabolism, Stilbenoid, diarylheptanoid, and gingerol biosynthesis, and other pathways (Supplementary Figure S2F).

#### 2.2.5. Overall Analysis of the Metabolome in DH40R versus DH199S

**Figure 3:**
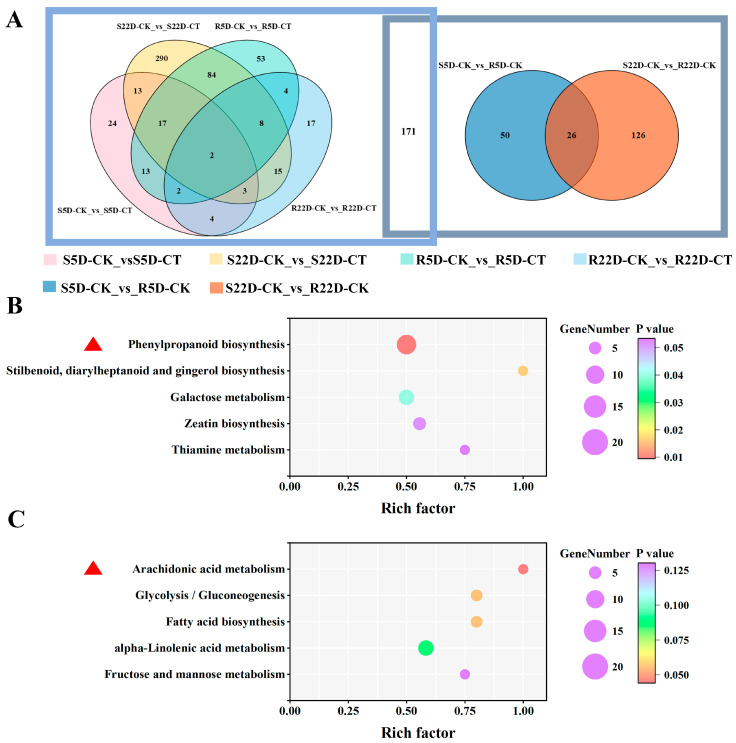
Venn diagrams and heatmaps among comparison groups and KEGG enrichment bubble charts for 257 and 248 metabolites. (**A**) Venn diagrams among S5D-CK_vs_S5D-CT, S22D-CK_vs_S22D-CT, R5D-CK_vs_R5D-CT, R22D-CK_vs_R22D-CT, S5D-CK_vs_R5D-CK, and S22D-CK_vs_R22D-CK. The rectangle on the left refers to the union of metabolites in these four groups (S5D-CK_vs_S5D-CT, S22D-CK_vs_S22D-CT, R5D-CK_vs_R5D-CT, R22D-CK_vs_R22D-CT). The rectangle on the right refers to the union of metabolites in these two groups (S5D-CK_vs_R5D-CK, and S22D-CK_vs_R22D-CK). (**B**) KEGG enrichment bubble charts for 257 metabolites. The red triangle indicates the pathway that was selected and discussed in detail. (**C**) KEGG enrichment bubble charts for 258 metabolites. The red triangle indicates the pathway that was selected and discussed in detail.

As shown in Figure 3A, a total of 580 DAMs were identified by comparing the metabolites of the *P. brassicae*-infected Chinese cabbage group with the control group and between the R and S lines. In the *P. brassicae*-infected materials, the accumulation levels of 549 metabolites were significantly different compared to the non-infected roots. Compared to the S line levels, 202 metabolites in the R line showed significant differences at all time points. These results were clearly illustrated in a Venn diagram. Among the 549 DAMs produced by *P. brassicae* infection, 384 (69.9%) responded to the infection at specific time points, while 165 (30.1%) responded at two or three time points. A total of 171 DAMs were not only differentially accumulated between the R and S lines but also varied following *P. brassicae* infection, especially the upregulated DAMs in the R line (R5D-CK_vs_R5D-CT and R22D-CK_vs_R22D-CT), totaling 257 DAMs that might have functions in clubroot resistance. In contrast, 248 DAMs identified only after clubroot infection in the S line (S5D-CK_vs_S5D-CT and S22D-CK_vs_S22D-CT) might be associated with the susceptible material’s response to clubroot. Certain metabolites showed a higher accumulation in the R line (Supplementary Figure S3). The most diverse metabolites were lignans and coumarins, followed by phenolic acids. To identify key metabolites related to clubroot resistance and susceptibility and to elucidate the molecular and material basis of resistance in different Chinese cabbage materials, we performed KEGG pathway analysis on these 257 and 248 metabolites treated at different times. As shown in Figure 3B, metabolites related to disease resistance were significantly enriched in phenylpropanoid biosynthesis, stilbenoid, diarylheptanoid and gingerol biosynthesis, and galactose metabolism, but especially in phenylpropanoid biosynthesis (*p* < 0.01). The 248 metabolites produced in response to clubroot in the S line were significantly enriched in arachidonic acid metabolism (*p* < 0.05) (Figure 3C).

### 2.3. Combined Analysis of Metabolomics and Transcriptomics

#### WGCNA Analysis of Genes Related to Phenylpropanoid Biosynthesis and Analysis of Arachidonic Acid Metabolism

**Figure 4:**
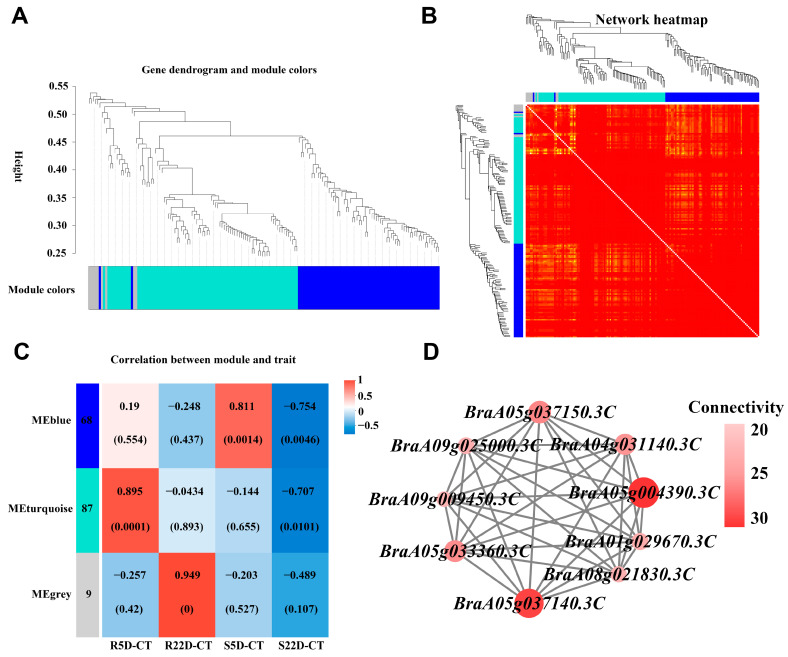
Co-expression analysis of genes related to phenylpropanoid− biosynthesis was conducted in the four groups (R5D-CT, R22-CT, S5D-CT, and S22D-CT). (**A**) Modular hierarchical clustering: The co-expression modules are depicted in different colors, while the gray modules indicate no correlation between genes. (**B**) Module gene clustering heat map. The gene expression network of phenylpropanoid biosynthesis-related genes in different tissues was analyzed using weighted gene co-expression network analysis, leading to the clustering of genes into distinct co-expression modules. (**C**) Module-to-sample correlation heat map. A correlation analysis was performed between the co-expression modules of various genes associated with phenylpropanoid biosynthesis in different tissues. The numbers above the heat map indicate the Pearson correlation coefficient (r) values. (**D**) Cytoscape representation of the co-expression network of the hub gene with kME > 0.8 in the MEturquoise module.

**Figure 5:**
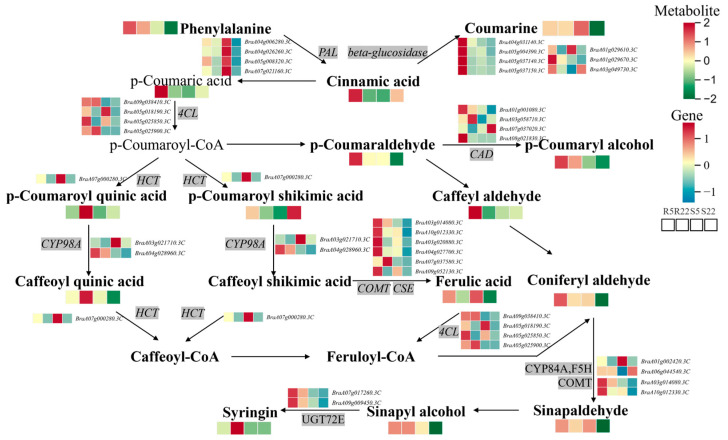
Profiling of transcripts and metabolites related to differentially expressed genes (DEGs) and differentially accumulated metabolites (DAMs) implicated in phenylpropanoid biosynthesis pathways in both “DH40R” and “DH199S” (“R5” means “R5D-CT”, “R22” means “R22D-CT”, “S5” means “S5D-CT”, and “S22” means “S22D-CT”).

To determine the complex expression relationships among genes related to phenylpropanoid biosynthesis in Chinese cabbage materials with different resistance levels and between inoculated and non-inoculated materials, we conducted a comprehensive weighted gene co-expression network analysis (WGCNA). This method grouped genes related to phenylpropanoid biosynthesis into two distinct WGCNA modules, and an additional unclustered gray module was observed (Figure 4A, Supplementary Table S3). The analysis of intra- and inter-module relationships showed strong correlations (Figure 4B). The turquoise module, composed of genes highly expressed in the resistant material DH40R, suggested that these genes played a crucial role in clubroot resistance (Figure 4C), with 87 genes in this module showing opposite expression patterns between the R and S lines. It was hypothesized that genes highly expressed in the early stages after infection in the R line greatly contributed to its resistance. Nine genes with high kME values (kME > 0.8), causing resistance–susceptibility differences, were identified in phenylpropanoid biosynthesis. To further determine the relationships between genes in the module and the screened hub genes (highly connected genes), we used the turquoise module to construct a protein interaction network related to differential expression in phenylpropanoid biosynthesis. In the turquoise module, nine genes related to phenylpropanoid biosynthesis had highly correlated expression, including *CAD* (*BraA08g021830.3C*), *4CL* (*BraA09g025000.3C*), *UGT72E* (*BraA09g009450.3C*), *beta-glucosidase* (*BraA05g037150.3C*, *BraA04g031140.3C*, *BraA05g004390.3C*, *BraA05g037140.3C*), *bglB* (*BraA01g029670.3C*), and *F6H1* (*BraA05g033360.3C*). These highly connected and interacting genes were considered central genes in the co-expression network and played a crucial role in understanding the biological mechanisms regulating phenylpropanoid biosynthesis (Figure 4D). We profiled transcripts and metabolites related to DEGs and DAMs within the phenylpropanoid biosynthesis pathways for both the “DH40R” and “DH199S” lines (where “R5” represents “R5D-CT”, “R22” represents “R22D-CT”, “S5” represents “S5D-CT”, and “S22” represents “S22D-CT”). In the R line, a higher accumulation of metabolites related to phenylpropanoid biosynthesis, including phenylalanine, p-coumaric acid, cinnamic acid, p-coumaraldehyde, p-coumaryl alcohol, p-coumaroyl quinic acid, caffeyl aldehyde, coniferyl aldehyde, and syringin, was observed compared to the S line. Numerous genes were upregulated or downregulated in the R line, suggesting that the substantial differences in metabolite accumulation and gene expression between the R and S lines were linked to their differential capabilities in defending against clubroot (Figure 5).

Similarly, by analyzing the metabolites in the S line, we screened out the arachidonic acid metabolism KEGG pathway that was most likely related to clubroot susceptibility. We analyzed the genes and metabolites in arachidonic acid metabolism (Supplementary Figure S4). After excluding low-expression genes, *PAL2G* (*BraA05g016980.3C*) was significantly upregulated at 22 DAI in the S line. At the same time, arachidonic acid was also significantly upregulated at 2 DAI in the S line. Combined with the phenotype of Chinese cabbage, we speculated that *PAL2G* may be a gene related to the disease susceptibility of Chinese cabbage after inoculation with *P. brassicae*. The upregulated expression of the metabolite arachidonic acid may induce plant tissue necrosis.

## 3. Discussion

Metabonomic analysis can improve the resistance of cabbage varieties to clubroot disease in breeding. Through correlation analysis between the phenotypic identification results of Chinese cabbage induced by *P. brassicae* and the concentration of metabolites, we can obtain the related metabolites indicating clubroot disease resistance. For materials that cannot be quickly identified as disease-resistant or susceptible, we can take the “concentration of metabolites” as a phenotype and associate it with genotype data to locate disease-resistant related gene loci. The contents of some metabolites closely related to clubroot resistance in different varieties of Chinese cabbage are determined, and then the whole genome sequencing of these Chinese cabbage is performed. The genotype of Chinese cabbage and phenotype of metabolites are analyzed. Genotyping-by-Sequencing (GBS) analyses of disease-resistance related metabolites are carried out in the F2 segregating population, and metabolomic quantitative trait loci (mQTLs) are carried out to verify the results and guesses of the metabolome genome-wide association study (mGWAS), and the function of candidate genes is verified by transgenic overexpression. Metabolites are the bridge between phenotype and genotype. These results provide a theoretical basis for the future Chinese cabbage breeding of resistance to clubroot.

Clarifying the mechanisms of resistance against clubroot disease in Chinese cabbage is key to refining integrated management measures. The objective of this study was to characterize the differential transcriptomic and metabolic responses induced by *P. brassicae* infection in the resistant variety DH40R and the susceptible variety DH199S. Although a previous study by our research group performed transcriptomic analysis on Chinese cabbage inoculated with *P. brassicae*, it did not comprehensively explore the metabolomic and transcriptomic differences between clubroot resistant and susceptible varieties. Thus, the present study filled this gap and further elucidated the disease-resistance mechanisms in Chinese cabbage after inoculation with *P. brassicae*.

After inoculation with *P. brassicae*, metabolites associated with *P. brassicae* infection were mainly enriched in secondary metabolism biosynthesis, such as phenylpropanoids, flavonoids, ubiquinones and other terpenoid quinones, phenylalanine, tyrosine, and tryptophan, in the resistant cultivar DH40R. These substances played crucial roles in the defense response of the Chinese cabbage cultivar DH40R against clubroot. Phenylpropanoid biosynthesis plays a vital role in the plant’s defense response against pathogens. Specific changes in several metabolites were associated with pathogen responses in susceptible cultivars. Metabolites related to pathogen infection in susceptible cultivars were mainly enriched in carbohydrate metabolism, ensuring the provision of the basic energy required for normal plant growth and defense responses.

### 3.1. Metabolite Response of Chinese Cabbage Inoculated with Plasmodiophora brassicae

After inoculation with *P. brassicae*, there was a notable alteration in metabolite expression in Chinese cabbage, involving significant upregulation or downregulation. Metabolic pathways involved in amino acid degradation are pivotal in plant defense mechanisms. Pipecolic acid, the degradation product of lysine, served as a key regulatory factor enabling disease resistance in plants. During drought conditions, the aspartic acid content increased in wheat [26]. Furthermore, there was a marked increase in the proline, histidine, isoleucine, and tryptophan contents in chickpea leaves [27]. Jasmonoyl-L-isoleucine and L-dihomomethionine were significantly upregulated in DH40R at 5 DPI with *P. brassicae*. Thus, we postulated that these two substances played a vital role in conferring disease resistance in Chinese cabbage.

Vitamins play pivotal roles in immunomodulation, with thiamine serving as a pivotal cofactor for numerous enzyme activities linked to the tricarboxylic acid cycle, pentose phosphate pathway, branched-chain amino acid pathway, anaerobic respiration, and pigment biosynthesis. Wang, et al. [28] demonstrated that thiamine enhances rice resistance to rice blast disease. Our findings showed that DAMs were enriched in thiamine metabolism in resistant lines during the early stage of infection, suggesting a close relationship between DH40R’s disease resistance and thiamine. There was significant upregulation of 11-cis-retinol and orotic acid levels in disease-resistant materials after inoculation, indicating a potential association between 11-cis-retinol, orotic acid, and plant disease resistance.

Nucleotides, as evolutionarily conserved compounds, played crucial roles in numerous biochemical reactions in plants, with their content frequently correlated with carbohydrate changes [29]. This study observed significant variations in nucleotide levels among materials and treatments, which served as essential energy substrates for diverse metabolic reactions.

In this study, a significant upregulation of flavonoids, including prunin, baimaside, and astragalin, and phenolic acids, including cinnamic acid, 4-hydroxy-3-methoxymandelate, hydrocinnamic acid, sinapyl alcohol, chlorogenic acid, p-coumaryl alcohol, and coniferyl alcohol, was observed in disease-resistant materials. The majority of these compounds have exhibited activity against pathogens. Prunin had demonstrated efficacy in inhibiting viral protein synthesis, reducing viral load, and decreasing the mortality rate of infected mice [30]. This compound possesses anti-inflammatory and antiviral properties and has been shown to be enriched in the callus tissues of peach anthers, where chlorogenic acid has also been observed [31]. The superoxide anion (O2•¯) is categorized as a reactive oxygen species (ROS), and its excessive accumulation induces oxidative damage in cells [32]. Astragalin is effective in treating various diseases, including cancer, diabetes, and obesity [33,34,35,36]. Cinnamic acid and its derivatives, particularly those containing phenolic hydroxyl groups, are potent antioxidants with antibacterial properties [37]. Plants bolster their defense against pathogens through hydrocinnamic acid accumulation [38]. 4-Hydroxy-3-methoxymandelate, commonly referred to as ferulic acid, was the predominant phenolic acid in plants and demonstrated robust antibacterial activity [39]. Research on sugarcane revealed a contrast in the synthesis rates of ferulic acid between resistant and susceptible lines, with the former demonstrating a heightened synthesis rate [40]. In addition to its direct antibacterial activity, ferulic acid also stimulates lignin synthesis, thereby indirectly defending against pathogen invasion. Ferulic acid exhibited its most potent resistance role during the initial plant growth stages [41]. Plant organisms harbored diverse forms of lignin and its constituent monomers. Lignin polymers in Poaceae plants predominantly consisted of three subunits: guaiacyl lignin, syringyl lignin, and p-hydroxyphenyl lignin. Sinapyl alcohol was predominantly polymerized into syringyl lignin, while coniferyl alcohol formed guaiacyl lignin. p-Coumaryl alcohol synthesized p-hydroxyphenyl lignin [42]. Following infection by *Fusarium oxysporum* f. sp. *albedinis*, resistant lines of *Phoenix dactylifera* L. mitigated pathogen-induced damage by enhancing cell wall lignification [43]. This study identified substantial disparities in the concentrations of diverse compounds, including amino acids, vitamins, nucleotides, carbohydrates, flavonoids, and phenolic acids, between disease-resistant and -susceptible materials. We hypothesized that these differences served as pivotal factors contributing to their phenotypic distinctions.

### 3.2. Metabolome and Transcriptome Analysis of the Response to P. brassicae Infection in Chinese Cabbage

*Plasmodiophora brassicae* infection induced differential metabolite accumulation in resistant and susceptible Chinese cabbage cultivars. To adapt to infection, the expression of nucleotides, amino acids, vitamins, flavonoids, and phenolic acids was upregulated in the resistant cultivar. These substances, which enhance resistance to *P. brassicae*, play a core role in maintaining the osmotic balance and scavenging ROS. Based on integrated metabolomics and transcriptomics analysis, we identified phenylpropanoid biosynthesis as the important KEGG pathway associated with disease resistance. *Phenylalanine ammonia-lyase* (*PAL*) catalyzes the deamination of L-phenylalanine to produce cinnamic acid. Furthermore, *p-coumarate 3-hydroxylase* (*C3H*) catalyzes the hydroxylation of pCA at the C3 position, leading to the formation of caffeic acid (CA), including hydroxylation reactions converting 4-Coumaroylshikimate into 5-O-Caffeoylshikimic acid and p-Coumaroyl quinic acid into 5-O-Caffeoylshikimic acid. *Caffeic acid/5-hydroxyferulic acid O-methyltransferase* (*COMT*) utilizes S-adenosyl-L-methionine as a methyl donor to methylate CA to ferulic acid (FA) and to convert 5-hydroxyferulic acid (5-OH-FA) to sinapic acid (SA). For example, *COMT* catalyzes the methylation of caffeic acid to produce ferulic acid and the conversion of 5-hydroxyferulic acid to SA. *Ferulic acid 5-hydroxylase* (*F5H*) catalyzes the hydroxylation of FA at the C5 position to form 5-hydroxyferulic acid (5OHFA). For example, *F5H* converts coniferyl aldehyde to 5-hydroxyconiferaldehyde and coniferyl alcohol to 5-hydroxyconiferyl alcohol. *4-Coumarate: CoA ligase* (*4CL*) catalyzes the conversion of trans-cinnamate to cinnamoyl-CoA. *Cinnamoyl-CoA reductase* (*CCR*) catalyzes the reduction of hydroxy-cinnamoyl CoA thioesters to their corresponding aldehydes. For example, *CCR* catalyzes the reduction of cinnamoyl-CoA to cinnamaldehyde and p-coumaroyl-CoA to p-coumaraldehyde. *Cinnamyl alcohol dehydrogenase* (*CAD*) catalyzes the final step of lignin precursor biosynthesis, including the reduction of caffeyl aldehyde to caffeyl alcohol, coniferyl aldehyde to coniferyl alcohol, 5-Hydroxyconiferaldehyde to 5-Hydroxyconiferyl alcohol, and sinapoyl aldehyde to sinapyl alcohol. In this study, genes showed differential expression during the early stages of *P. brassicae* infection, which correlated with alterations in metabolites with antimicrobial and disease-resistant properties in both resistant and susceptible materials. Thus, we hypothesized that the disease resistance observed in resistant materials was closely associated with phenylpropanoid biosynthesis. Wang, et al. [44] conducted integrated transcriptomic and metabolomic analysis on two rice varieties differing in disease resistance, revealing a potential correlation between phenylpropanoid biosynthesis and Al resistance in disease-resistant varieties. Zhou, et al. [45] performed integrated metabolomic and transcriptomic analysis on blister blight disease-resistant tea plants and found that the majority of DEGs were significantly enriched in plant–pathogen interaction and phenylpropanoid biosynthesis pathways, offering comprehensive insights into the resistance mechanism in tea plants against blister blight disease. Xing, et al. [46] identified the crucial role of PAL-related genes in inducing resistance against anthracnose in 84K poplar. Tang, et al. [47] employed non-targeted metabolomics to analyze tomatoes inoculated with *Cryptococcus laurentii* and revealed that phenylpropanoid biosynthesis pathway activation and subsequent metabolite changes were crucial factors in inducing resistance. Xu, et al. [48] demonstrated that *OsGRP3* enhanced drought resistance in rice by modulating the phenylpropanoid biosynthesis pathway and promoting lignin accumulation. Ling, et al. [49] performed omics analysis on two melon (*Cucumis melo* L.) varieties and found that both varieties suppressed flavonoid biosynthesis by downregulating related genes and promoting lignin production, offering unique insights into the defense mechanisms in melon against downy mildew. Xue, et al. [50] demonstrated that bacillomycin D-C16 activated phenylpropanoid biosynthesis, thereby enhancing disease resistance in cherry tomatoes. Through transcriptomic analysis, different KEGG pathways were enriched in clubroot-resistant and -susceptible Chinese cabbage varieties, and a large number of genes were differentially expressed in these pathways [12,18,19,51,52,53,54,55].

Wang, et al. [56] pointed out that the arachidonic acid (AA) pathway plays a key role in cardiovascular biology, carcinogenesis, and many inflammatory diseases, such as asthma and arthritis. Dedyukhina, et al. [57] found that AA acted as an inducer in defensive responses in plants against phytopathogens. The inducing activity of AA largely depends on its concentration. High AA concentrations induce tissue necrosis and antimicrobial compound (phytoalexins) accumulation in plants, while low concentrations lead to long-term resistance to phytopathogen infections, similar to an immunological process. Garcı, et al. [58] noted that arachidonic acid was an effective inducer of programmed cell death and defense responses in Solanaceae plants. The fatty acid structure influenced cellular activities through membrane lipid changes and bioactive derivative generation. Eicosapolyenoic acids released during oomycete infection activate plant defenses. Transgenic *Arabidopsis thaliana* plants (EP) producing eicosadienoic, eicosatrienoic, and arachidonic acid mimic this response, enhancing resistance against fungal, oomycete, and insect pathogens but increasing susceptibility to bacteria. Elevated jasmonic acid and reduced salicylic acid levels in EP plants indicated the modulation of stress-responsive gene networks by eicosapolyenoic acids, with arachidonic acid as a key signaling molecule promoting stress gene expression and reducing *Botrytis cinerea* susceptibility in tomato leaves [59]. Differences in the genes and metabolites related to phenylpropanoid biosynthesis and arachidonic acid metabolic pathways have also been observed, suggesting that these metabolites and genes are closely associated with differences in disease resistance.

## 4. Materials and Methods

### 4.1. Materials, Growth Conditions, and Test Strains

The DH lines DH40 and DH199 were used in this study and acquired via microspore culture from the Institute of vegetables, Henan Academy of Agricultural Sciences. All Chinese cabbage materials in the metabolome can be divided into eight groups: R5D-CK, R5D-CT, R22D-CK, R22D-CT, S5D-CK, S5D-CT, S22D-CK, and S22D-CT. Each group has three biological replicates. “CT” refers to inoculated samples, while “CK” refers to uninoculated controls. “R” represents the resistant material DH40, and “S” represents the susceptible material DH199 (Supplementary Table S4). The tested strain was obtained from *P. brassicae*-infected Chinese cabbage in Xinye County, Henan Province, and identified as pathogen isolate 4, indicating its higher pathogenicity. The strain was characterized using the ECD differentiation system and categorized as ECD21/31/31 [54].

Seeds from two Chinese cabbage DH lines (DH40 and DH199) were germinated in distilled water for 3 days and transplanted to 50-well plates with a sterile substrate. The roots were inoculated with *P. brassicae* in a growth chamber at 20–25 °C with a 16 h light/8 h dark photoperiod and watered daily.

Root nodules stored in a −20 °C freezer were thawed, weighed, crushed, filtered, and centrifuged; the volume was adjusted to a 1:10 ratio with sterile water. *Plasmodiophora brassicae* was activated overnight and measured, and its concentration was adjusted to 1–10 × 10^7^ CFU/mL. Then, 4 mL of the *P. brassicae* suspension was injected into the roots of seedlings, which were then incubated in the dark to promote infection [54,60].

Microscopic examination of *P. brassicae* infection in resistant (DH40R) and susceptible (DH199S) materials revealed that the root hairs of both DH199S and DH40R were clean, with no clubroot pathogens observed before inoculation. By 5 DPI, a small number of zoosporangia and secondary zoospores were observed in the cortical cells of DH40R, whereas DH199S exhibited a large number of zoosporangia and secondary zoospores in its cortical cells. By 22 DPI, DH40R showed no symptoms, while DH199S was completely infected, reaching a disease severity level of 7. Paraffin sectioning revealed a few immature zoospores in the cortical cells of DH40R, which showed normal development. DH199S had abnormally swollen cortical cells with significantly increased cell numbers, and its cortical cells were filled with mature zoosporangia and resting spores, leading to a large number of abnormally swollen and dividing cortical cells compressing the vascular tissue and causing irregular swelling in root tumors on the main root, with small spherical tumors also presented on the lateral roots [60].

### 4.2. Metabolomics

#### 4.2.1. Sample Extraction Procedure

The materials underwent vacuum freeze-drying in a freeze-dryer (Scientz-100F, Ningbo, China) and were subsequently ground to a powder using a grinder (MM 400, Retsch, Haan, Germany) operating at 30 Hz for 1.5 min. A 100 mg sample of the ground powder was weighed and dissolved in 1.2 mL of 70% methanol extraction solution. The samples were vortexed 6 times for 30 s every 30 min and subsequently stored overnight at 4 °C. After centrifuging the samples at 12,000 rpm for 10 min, the supernatant was collected, filtered through a 0.22 μm microporous membrane, and transferred to sample vials for subsequent UPLC-MS/MS analysis.

#### 4.2.2. Chromatography Mass Spectrometry Acquisition Conditions

The data acquisition system primarily consisted of an Ultra Performance Liquid Chromatography (UPLC) unit (SHIMADZU Nexera X2, https://www.shimadzu.com.cn/ (accessed on 21 March 2024)) and a Tandem Mass Spectrometry (MS/MS) device (Applied Biosystems 4500 QTRAP, https://www.thermofisher.cn/cn/zh/home/brands/applied-biosystems.html (accessed on 22 March 2024)).

The liquid phase conditions were as follows: (1) Column: Agilent SB-C18 1.8 µm, 2.1 mm × 100 mm; (2) Mobile phase: Phase A was ultra-pure water (with 0.1% formic acid), Phase B was acetonitrile (with 0.1% formic acid); (3) Gradient: Starting at 0.00 min, the proportion of Phase B was 5%, reaching 95% within 9.00 min, maintained for 1 min, decreased to 5% from 10.00 to 11.10 min, and maintained at 5% for 14 min; (4) Flow rate: 0.35 mL/min; Column temperature: 40 °C; Injection volume: 4 μL.

The primary conditions for mass spectrometry included acquiring the LIT and triple quadrupole (QQQ) scanned on a triple quadrupole linear ion trap mass spectrometer (Q TRAP), specifically the AB4500 Q TRAP UPLC/MS/MS system equipped with an ESI Turbo ion spray interface. The system was controlled by Analyst 1.6.3 software (AB Sciex) for operation in both the positive and negative ion modes. The operating parameters of the ESI source were as follows: ion source, turbo spray; source temperature set to 550 °C; ion spray voltage (IS) at 5500 V (positive ion mode)/−4500 V (negative ion mode); ion source gases I (GSI), II (GSII), and curtain gas (CUR) set at 50, 60, and 25.0 psi, respectively. The collision-induced ionization parameters were set to high. Instrument tuning and mass calibration were performed using 10 and 100 μmol/L polyethylene glycol solutions in QQQ and LIT modes, respectively. QQQ scans were performed in MRM mode with collision gas (nitrogen) set to medium. For each MRM ion pair, DP and CE were further optimized, and a specific set of MRM ion pairs was monitored for each period based on the metabolites eluted within that period.

#### 4.2.3. Quality Control Samples

Quality control (QC) samples were prepared by combining extracts from Chinese cabbage root samples to evaluate the sample repeatability under consistent treatment conditions. In the instrument analysis, a QC sample was included for every 10 test samples to ensure the repeatability of the results.

#### 4.2.4. Data Processing and Analysis

The metabolites were analyzed using the MetWare UPLC-MS/MS detection platform and processed using Analyst 1.6.3 software for qualitative and quantitative analysis. This included peak detection, filtering, and alignment to obtain corresponding peak areas and the relative content. Qualitative substance analysis was performed using the MetWare self-built MWDB (MetWare database), utilizing secondary spectrum information and removing redundant signals, such as isotope signals and duplicate signals of ions (e.g., K^+^, Na^+^, and NH^4+^) and fragment ions of larger molecules. PCA, orthogonal partial least squares-discriminant analysis, and hierarchical cluster analysis were performed using R software (R version 3.5.1) to analyze the overall and within-group accumulation patterns of the metabolites. Substances with an RSD > 30% in QC samples were filtered out during data quality control. Differential metabolites for each group were selected based on the significance threshold (*p*-value), variable importance in projection (VIP), and fold change (FC) and annotated and analyzed for metabolic pathways using the KEGG database.

### 4.3. Transcriptomics

Total RNA was extracted, checked for purity and integrity, and used for cDNA synthesis. The library was prepared by synthesizing first- and second-strand cDNA, purifying fragments, and performing terminal repair and PCR. The libraries were sequenced on the Illumina HiSeq platform, producing 150 bp paired-end reads. Quality control removed adapters and low-quality reads, yielding clean data. Bowtie2 indexed the genome, and HISAT2 aligned the clean data to the *B. rapa* genome v3.0 (http://www.brassicadb.cn/#/Download/ (accessed on 1 May 2020)) [61,62,63].

The expression levels of three biological replicates between the two samples were calculated using DESeq2 v 1.0 software. Genes with |log_2_ fold change| ≥ 1 and a false discovery rate (FDR) adjusted *p*-value (q-value) < 0.05 were selected as DEGs. KEGG pathway analysis was performed, and heatmaps were generated using the Omicshare platform (www.omicshare.com/tools (accessed on 9 July 2024)). We used R software to carry out WGCNA analysis [64]. The interaction network diagram of genes in modules was visualized using Cytoscape3.8.2 software.

Total RNA was extracted using the Takara MiniBEST Plant RNA Extraction Kit (Takara, Dalian, China). Reverse transcription was performed using the RevertAid First Strand cDNA Synthesis Kit (Takara), and qRT-PCR was conducted as previously described. GAPDH was used as the internal reference gene for Chinese cabbage [64]. The transcription levels of the DEGs were detected using qRT-PCR. The relative expression levels of the target genes were calculated using the 2^−△△Ct^ method [65].

The transcriptome data can be found in the NCBI Short Read Archive (SRA) database under the accession number PRJNA692311 [54]. For portions of the data utilized in this article from that source, see Supplementary Table S5.

## Data Availability

The transcriptome data can be found in the NCBI Short Read Archive (SRA) database under the accession number PRJNA692311.

## Supplementary Materials

The following supporting information can be downloaded at https://www.mdpi.com/article/10.3390/ijms251910440/s1.
