# Supplementary figures and images for: Comparative Metabolome and Transcriptome Analysis Reveals the Defense Mechanism of Chinese Cabbage (Brassica rapa L. ssp. pekinensis) against Plasmodiophora brassicae Infection
Source: Int J Mol Sci. 2024 Sep 27;25(19):10440. doi: 10.3390/ijms251910440 (PMC11476981; doi:10.3390/ijms251910440)

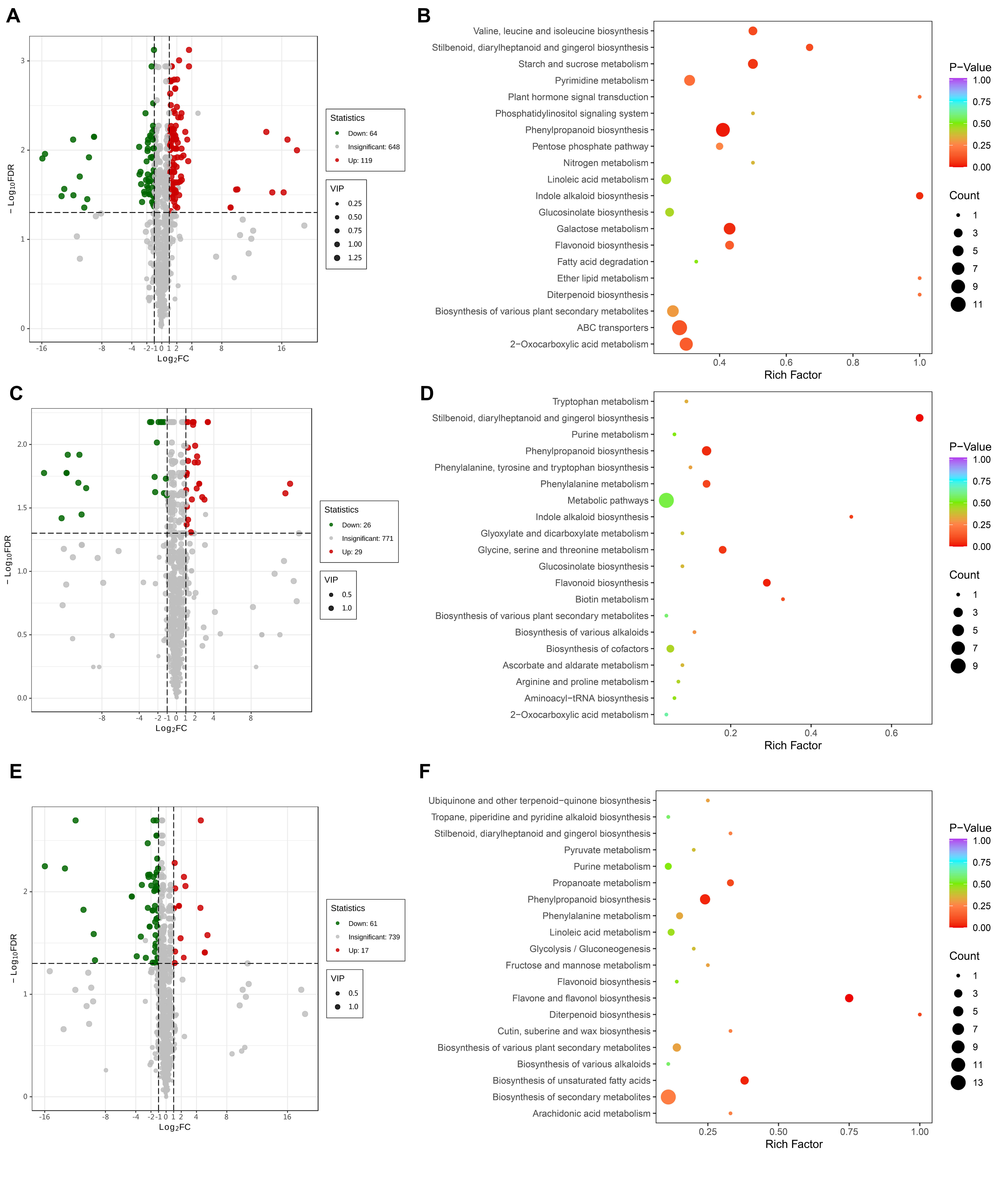

Supplement: Supplementary file 1 [file ijms-25-10440-s001.zip › 2024.09.26.Supplementary materials revision/Supplementary Figure S1.tif]

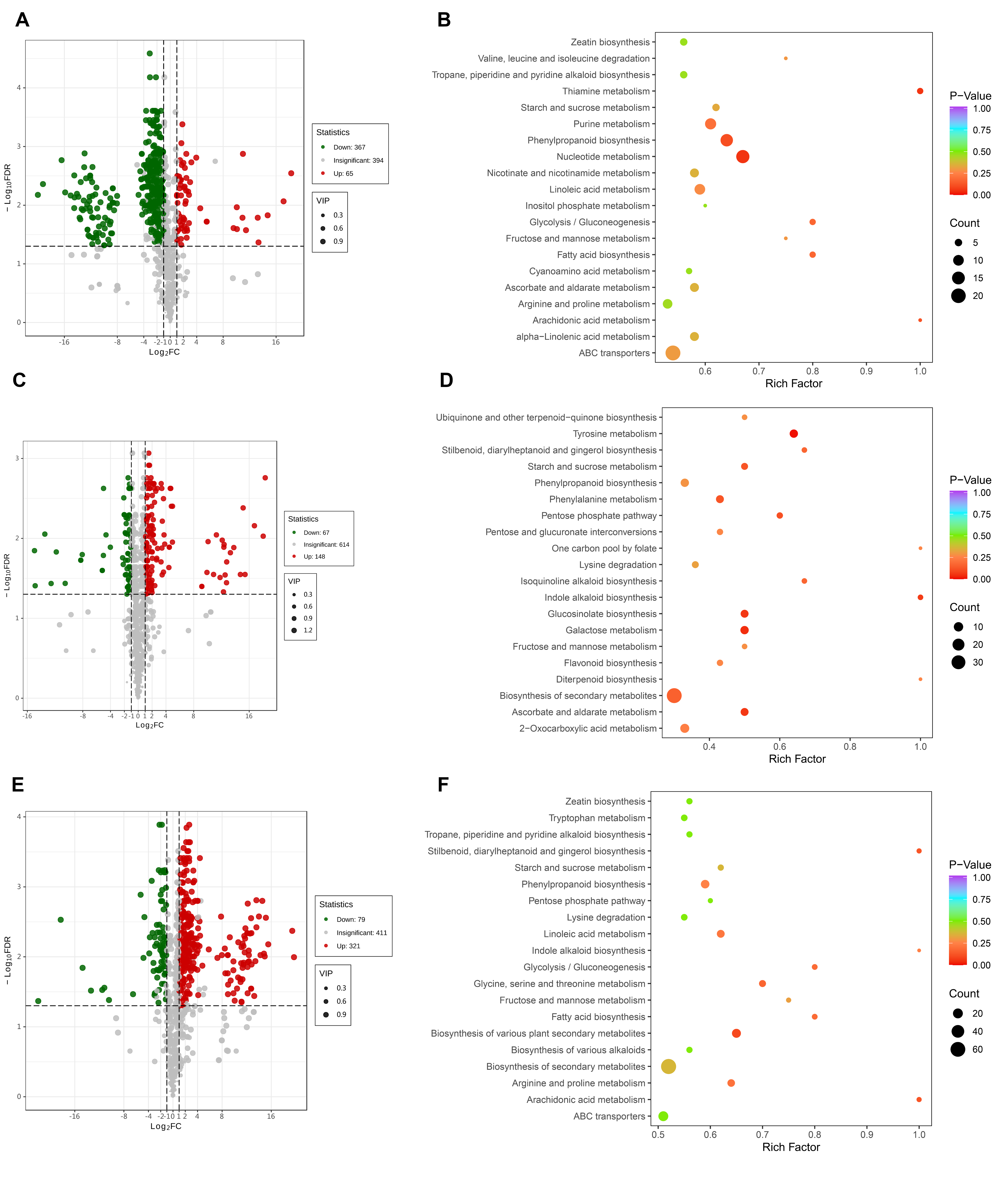

Supplement: Supplementary file 1 [file ijms-25-10440-s001.zip › 2024.09.26.Supplementary materials revision/Supplementary Figure S2.tif]

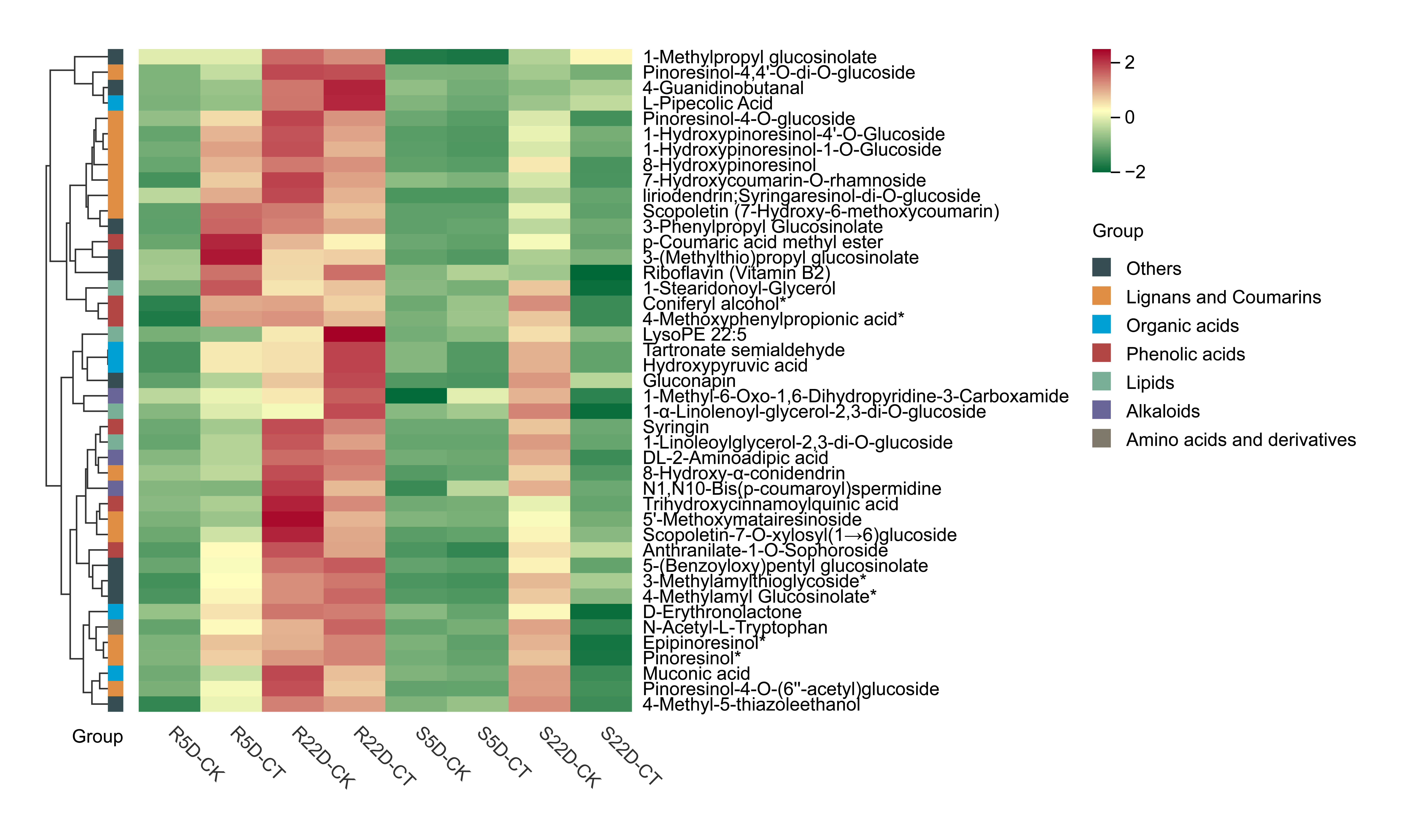

Supplement: Supplementary file 1 [file ijms-25-10440-s001.zip › 2024.09.26.Supplementary materials revision/Supplementary Figure S3.tif]

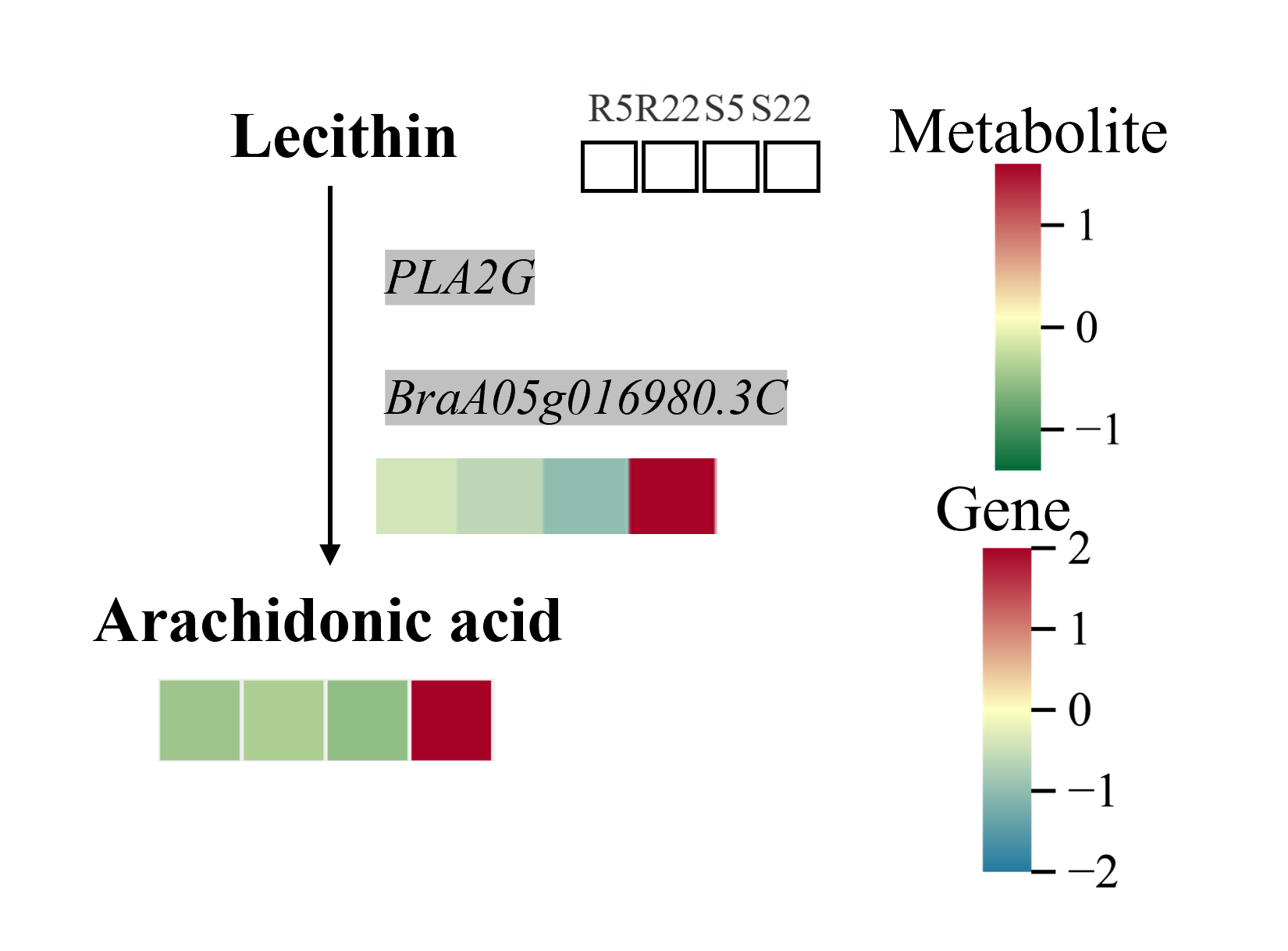

Supplement: Supplementary file 1 [file ijms-25-10440-s001.zip › 2024.09.26.Supplementary materials revision/Supplementary Figure S4.tif]
